# Remodelling of corticostriatal axonal boutons during motor learning

**DOI:** 10.1038/s41586-025-09336-w

**Published:** 2025-07-30

**Authors:** Mengjun Sheng, Di Lu, Richard H. Roth, Fuu-Jiun Hwang, Kaiwen Sheng, Jun B. Ding

**Affiliations:** 1 Department of Neurosurgery, Stanford University School of Medicine, Stanford, CA USA; 2 Aligning Science Across Parkinson’s (ASAP) Collaborative Research Network, Chevy Chase, MD USA; 3 Stanford Bioengineering PhD program, Stanford University, Stanford, CA USA; 4 Department of Neurology and Neurological Sciences, Stanford University, Stanford, CA USA; 5 The Phil and Penny Knight Initiative for Brain Resilience at the Wu Tsai Neurosciences Institute, Stanford University, Stanford, CA USA

**Keywords:** Basal ganglia, Spine plasticity

## Abstract

Motor skill learning induces long-lasting synaptic plasticity at dendritic spines^1–4^ and at the outputs of motor cortical neurons to the striatum^5,6^. However, little is known about corticostriatal axon activity and structural plasticity during learning in the adult brain. Here, using longitudinal in vivo two-photon imaging, we tracked thousands of corticostriatal axonal boutons in the dorsolateral striatum of awake mice. We found that learning a new motor skill dynamically regulated these boutons. The activities of motor corticostriatal axonal boutons exhibited selectivity for rewarded movements (RM) and unrewarded movements (UM). Notably, boutons on the same axonal branches showed diverse responses during behaviour. Motor learning significantly increased the proportion of RM boutons and reduced the heterogeneity of bouton activities. Moreover, motor learning induced profound structural dynamism in boutons. By combining structural and functional imaging, we saw that newly formed axonal boutons were more likely to exhibit selectivity for RM and were stabilized during motor learning, whereas UM boutons were selectively eliminated. These findings reveal a novel form of plasticity in corticostriatal axons and show that motor learning drives dynamic bouton reorganization to support motor skill acquisition and execution.

## Abstract (web summary)

Individual boutons on mouse corticostriatal axons exhibit differential selectivity for rewarded and unrewarded movements during motor learning, and the proportion of reward-responsive boutons increases.

## Main

Learning and executing fine movement skills require corticostriatal circuits^7–10^. During motor learning, neuronal ensembles in the primary motor cortex (M1) first expand and later refine it into a smaller population that generates reproducible activity sequences^8,11^. M1 neurons project to the dorsolateral striatum (DLS)^12–14^ and drive striatal spiny projection neurons (SPNs)^15^. DLS activity reflects that of M1 neurons^15^ and reorganizes to encode movement sequences^16^. Motor learning also remodels dendritic spines, strengthening synaptic connections to the striatum^6^. Conversely, spine loss in disorders such as Parkinson’s disease disrupts corticostriatal transmission^17–19^. Although spine plasticity is well-characterized, whether presynaptic boutons also undergo in vivo activity and structural remodelling remains unclear^20–23^.

**Fig. 1:**
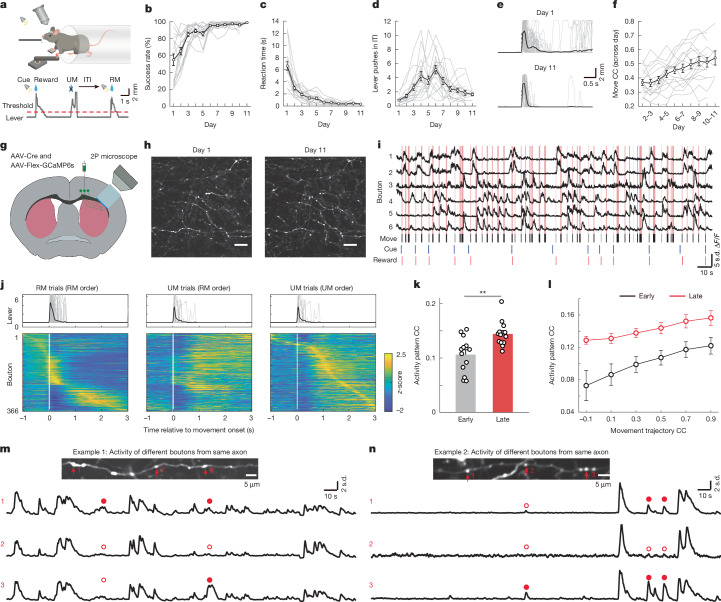
Longitudinal two-photon Ca^2+^ imaging of corticostriatal axonal boutons during motor learning. **a**, Schematic of lever-pushing task in which mice received water rewards following a cue. Example shows two rewarded (RM) and one unrewarded (UM) movement during the ITI. **b**–**d**, Behavioural improvements over training (*n* = 17 mice): increased success rate (**b**), decreased reaction time (**c**) and reduced movements during the ITI (**d**). Grey lines represent individual mice and the black line shows the group average. **e**, Representative RM trajectories on day 1 and day 11 from one mouse. Grey lines represent single trials, black line shows the average and the red dotted line dashed line indicates movement onset. **f**, Movement trajectories became more consistent across trials during training (*r* = 0.44, *P* = 1.05 × 10^−9^, Pearson’s correlation). CC, cross-correlation. **g**, Schematic of viral injection in M1 and imaging in DLS. 2P microscope, two-photon microscope. **h**, Example GCaMP6s-labelled corticostriatal axons on day 1 and 11. Scale bars, 20 μm. **i**, Task-related activity traces from boutons on day 3. Red lines indicate movement, black bars show lever pushes, blue represents cue and red represents reward. **j**, Top, individual (grey) and average (black) RM and UM trajectories. Bottom, averaged activity of 426 boutons aligned to RM or UM onset; boutons sorted by peak activity time. **k**, Increased trial-to-trial bouton activity correlation during RM in late versus early learning (**P = 0.007, Wilcoxon rank sum test, *n* = 13 mice). **l**, Trial-to-trial activity correlation plotted against movement similarity shows stronger coupling over learning (repeated measures two-way ANOVA, Bonferroni post hoc correction, *P* < 0.001 at multiple bins). **m**,**n**, Top, averaged bouton images from example axons 1 (**m**) and 2 (**n**). Bottom: Δ*F*/*F*_0_ traces from three boutons with bouton-specific Ca^2+^ events, including detected events (filled dots) and the corresponding absences (open dots). Error bars indicate s.e.m. Source Data

We trained mice to perform a cued lever-pushing task under a two-photon microscope^16^ (Fig. 1a). Lever pushes beyond a set threshold after cue onset were rewarded with water; uncued pushes during the inter-trial interval (ITI) were not rewarded and triggered an additional timeout (Supplementary Video 1). Mice were trained daily for approximately two weeks (*n* = 17 mice). Over time, success rate (Fig. 1b) and reaction times (Fig. 1c) improved, and ITI lever pushes decreased (Fig. 1d and Extended Data Table 1). Lever trajectories also became more stereotyped (Fig. 1e), with higher pairwise correlation between trials (Fig. 1f)—a signature of motor learning^8,11,16^.

## Movement-related M1 axonal bouton activities

To investigate bouton activity during motor learning, we injected adeno-associated virus (AAV) encoding the genetically encoded Ca^2+^ indicator GCaMP6s^24^ into M1 layer 5^25^ and implanted a chronic window above the DLS (Fig. 1g). After recovery, we performed longitudinal two-photon calcium imaging while monitoring behaviour (Fig. 1h and Supplementary Video 2). Activity in individual M1 boutons strongly correlated with lever movements (Fig. 1i). The activity of individual boutons spanned over the entire duration of the RM (Fig. 1j, left), consistent with known M1 somatic patterns^11^. During UMs, bouton ensemble activity differed in temporal structure (Fig. 1j, middle), but reordering boutons by their UM peak timing revealed preserved sequential activity (Fig. 1j, right). Consistent with previous reports^11^, the pairwise correlation on trial-to-trial activity significantly increased in the late stages of motor learning compared with that of the early stages (Fig. 1k, early stage: day 1 to day 3; late stage: ≥day 8). We further evaluated the relationship between movement and axonal bouton activity during early and late stages of learning. By sorting trials according to the similarity of movements for each pair of trials, we found the overall activity pattern pairwise correlation was significantly higher in the late stage compared with the early stage, even when mice generated dissimilar movement trajectories (Fig. 1l), suggesting that the overall bouton activity pattern became more consistent at late sessions.

Cortical axons arborize extensively in the striatum and form en passant synapses^26,27^. We identified boutons from the same axon and examined their Ca^2+^ activity (Fig. 1m,n). Because of the high fidelity of action potential propagation along the axons^28–30^, multiple release sites at these en passant synapses are thought to deliver cortical outputs faithfully to multiple postsynaptic striatal neurons. Unexpectedly, we found bouton-level heterogeneity—some boutons showed unique transients not shared by others on the same axon (Fig. 1m,n and Supplementary Video 3). These results suggest that the activity of M1 corticostriatal axonal boutons is movement-related and can be modulated by reward. Furthermore, the boutons formed on the same axons exhibit heterogeneous activity patterns.

## Reward modulation of movement bouton activities

**Fig. 2:**
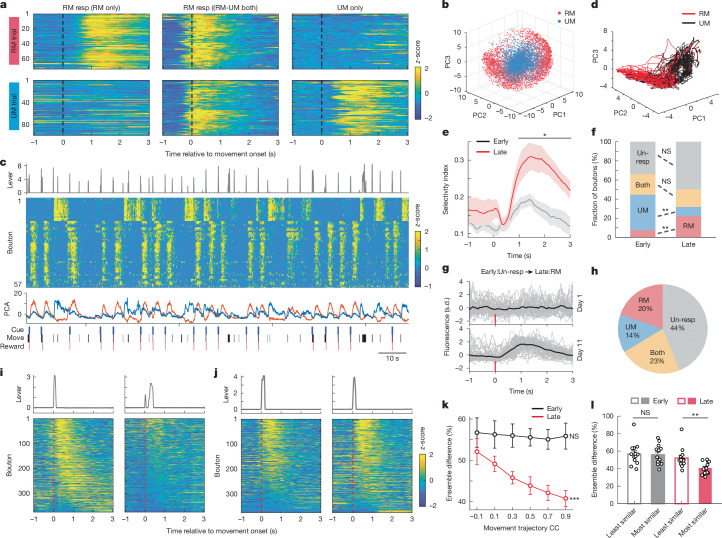
Reward- and movement-related activity of M1 corticostriatal boutons. **a**, Example peri-movement activity of three boutons during RM (top) and UM (bottom) trials. Left, RM-selective bouton. Middle, RM–UM both. Right, UM-selective bouton. Resp, responsive. **b**, PCA embedding of all boutons (*n* = 3,744 RM, *n* = 4,211 UM, 8 mice). RM-only (red) and UM-only (blue) boutons are distinct. **c**, Top, lever movement trajectory. Second row, activity of 57 boutons (15 UM and 42 RM) from one mouse. Each row represents one bouton. Third row, PC1 (orange) and PC2 (blue) of bouton population activity. Bottom, behavioural annotations: cue, movement (move) and reward. **d**, 3D PCA trajectories of neural activity for RM and UM trials from one representative session. **e**, Trajectory selectivity index for RM versus UM trials at early and late learning stages (*P* < 0.05, Wilcoxon rank sum test, *n* = 8 mice). Shaded areas represent s.e.m. **f**, Change in bouton reward selectivity during motor learning. RM: *P* = 0.003, UM: *P* = 0.0011, both: *P* = 0.96, un-resp: *P* = 0.19; Wilcoxon rank sum test, *n* = 8 mice. Un-resp, unresponsive. **g**,**h**, Example of a bouton gaining RM selectivity (**g**) and the fate of early UM boutons over learning (**h**). **i**,**j**, Example trials with dissimilar (**i**) and similar (**j**) movement trajectories and corresponding bouton ensemble activity. **k**, Ensemble activity difference negatively correlates with movement similarity in late stage (*r* = −0.46, *P* = 1.89 × 10^−5^), but not early (*r* = −0.04, *P* = 0.73; Pearson’s correlation, *n* = 13 mice) learning. **l**, Ensemble differences between trials with most similar or least similar movement trajectories (early: *P* = 0.88, late: *P* = 0.0035, two-sided Wilcoxon rank sum test, *n* = 13 mice). *P < 0.05, **P < 0.01, ***P < 0.001; NS, not significant. Error bars represent s.e.m. Source Data

To assess how reward modulates bouton activity, we aligned GCaMP6s signals to RM or UM trial onsets. Some boutons showed activity only during RM trials (Fig. 2a, left); some showed activity only during UM trials (Fig. 2a, right), and others showed activity during both (Fig. 2a, middle). We classified the boutons into three categories: RM-only, UM-only and ‘RM–UM both’ boutons. For some analyses, RM-only and RM–UM both boutons were grouped as RM-responsive. To validate this classification, we performed a principal components analysis (PCA) on bouton activity during RM and UM trials. Boutons were embedded into 3D PC space using the first three components. RM-only and UM-only boutons formed distinct clusters (Fig. 2b), indicating that activity patterns differ between reward conditions.

We next examined the population bouton activity of RM-only and UM-only boutons in consecutive trials, plotted the amplitude of principal component over time, and aligned with movement behaviour (Fig. 2c). Further analysis of principal component trajectories revealed that principal components 1 and 2 (PC1 and PC2) captured RM- and UM-related responses, respectively. Activity trajectories in principal component space were also distinct between RM and UM trials (Fig. 2d). Notably, the selectivity index separating RM and UM trajectories significantly increased during late learning, indicating enhanced bouton selectivity for reward outcomes (Fig. 2e).

**Extended Data Fig. 1:**
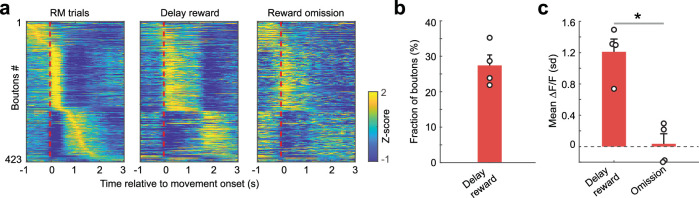
Corticotriatal axonal activities during RM, reward delay and reward omission trials. **a**, Averaged activity pattern of same population of boutons during RM, delay reward and reward omission trials (423 boutons from one example mouse). **b**, Fraction of delay reward modulated boutons (n = 4 mice). **c**, Mean activity of delay reward modulated boutons during delay reward and reward omission trials (P = 0.029, two-sided Wilcoxon rank sum test, n = 4 mice). *p < 0.05. Error bars represent SEM. Source Data

**Extended Data Fig. 2:**
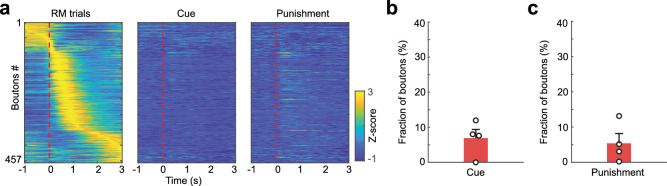
Corticostriatal axonal bouton responses to cue and punishment. **a**, Averaged activity pattern of same population of boutons during RM, cue and punishment (457 boutons from one example mouse). **b**, Fraction of cue modulated boutons (n = 4 mice). **c**, Fraction of punishment modulated boutons (n = 4 mice). Error bars represent SEM. Source Data

**Extended Data Fig. 3:**
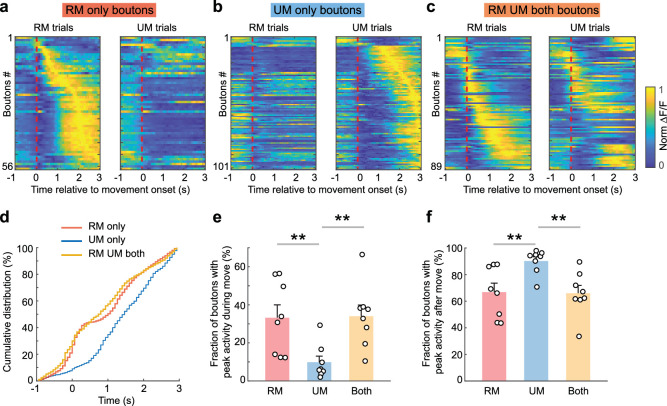
Averaged activity pattern of RM only, UM only and RM UM both boutons. **a**, Averaged activity pattern of RM only boutons during RM trials (left) and UM trials (right), sorted according to the activity peak time in RM trials (one example mouse). **b**, Averaged activity pattern of UM only boutons during RM trials (left) and UM trials (right), sorted according to the activity peak time in UM trials (one example mouse). **c**, Averaged activity pattern of RM UM both boutons during RM trials (left) and UM trials (right), sorted according to the activity peak time in RM trials (one example mouse). **d**, Cumulative distribution of activity peak time of RM only (red), UM only (blue) and RM UM both (yellow) boutons (n = 8 mice). **e**, Fraction of boutons with activity peaks during the lever movement period (peak at −0.4 s to 0.5 s, n = 8 mice, RM vs UM, P = 0.0047, UM vs Both, P = 0.0019, two-sided Wilcoxon rank sum test). **f**, Fraction of boutons with activity peaks after the lever movement (peak at >0.5 s, n = 8 mice, RM vs UM, P = 0.0047, UM vs Both, P = 0.0019, two-sided Wilcoxon rank sum test). **p < 0.01. Error bars represent SEM. Note that more RM only and RM UM both boutons activated during lever pushing period, while more UM only boutons activated after lever pushing. Source Data

To dissociate whether bouton activity encoded movement or reward, we introduced reward delay and omission trials during imaging. In reward delay trials, a subset of RM boutons (27.4% ± 5.9%) shifted their peak activity to coincide with delayed reward (Extended Data Fig. 1a,b); These boutons were inactive during reward omission trials (Extended Data Fig. 1c), suggesting that this subset of RM boutons was modulated by reward rather than movement. In cue-only and punishment-only trials, small fractions of RM boutons were modulated by either cue (7.8% ± 5.7%; Extended Data Fig. 2a,b) or punishment (7.3% ± 6.7%; Extended Data Fig. 2c). Furthermore, we plotted the activity profiles for RM-only, UM-only and RM–UM both boutons in RM and UM trials, these three types of boutons all exhibited firing patterns with activity during and after lever pushing (Extended Data Fig. 3a–c). When comparing peak activity timing, RM-only and RM–UM both boutons were active earlier than UM-only boutons (Extended Data Fig. 3d). Together, these results highlight that most RM boutons encode movement, but a subset is modulated by the reward, cue or punishment.

The emergence of more consistent activity patterns of corticostriatal boutons in late sessions may result from reward-based reinforcement of certain activity–reward outcome pairs out of initial exploration during learning. In this case, the activity of RM-only or UM-only boutons during the early stage may have a similar representation at the late stage. Alternatively, the learned activity pattern may require dynamic rearrangement of bouton ensembles, which may result in changes in the representation of RM or UM.

**Extended Data Fig. 4:**
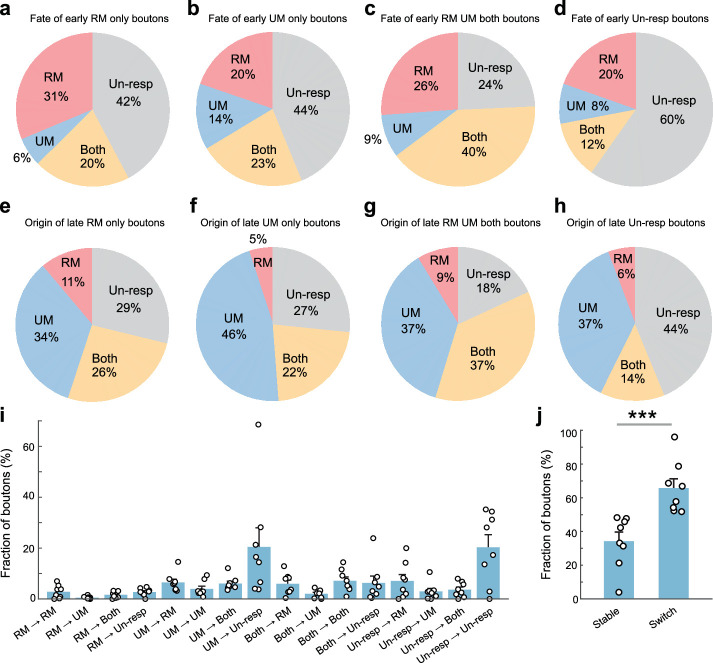
Dynamic changes of bouton selectivity during motor learning. **a-d**, Fate of classified RM only (**a**), UM only (**b**), RM-UM both (**c**) and Un-responsive (**d**) boutons identified by their responses during early stages of training (n = 8 mice). **e-h**, Origin of classified RM only (**e**), UM only (**f**), RM-UM both (**g**) and Un-responsive (**h**) boutons identified by their responses during late stages of training (n = 8 mice). **i**, Fraction of boutons for 16 types defined by their dynamic change (n = 8 mice). Error bars represent SEM. **j**, Fraction of stable and switching boutons. Stable boutons represent the boutons that were classified as the same types at the early and late stages, while switching boutons represent boutons that were classified as different types at the early and late stages (P = 1.6 × 10^−4^, two-sided Wilcoxon rank sum test, n = 8 mice). ***P < 0.001, error bars represent SEM. Source Data

**Extended Data Fig. 5:**
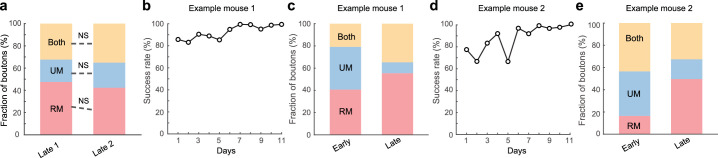
Stability of bouton representation during motor learning. **a**, RM UM representation on two different days at late stage of learning (P = 0.8, P = 1, P = 0.8 respectively for RM, UM and RM-UM both, two-sided Wilcoxon rank sum test, n = 6 mice). **b-e**, Reward representation for 2 mice with good behavior performance at the early stage of learning. **b**, Success rate of example mouse 1 across training days. **c**, Change of RM UM representation during motor learning for mouse 1. **d**, Success rate of example mouse 2 across training days. **e**, Change of RM UM representation during motor learning for mouse 2. Source Data

We next explored how bouton representations of reward outcomes change with learning. Across early and late sessions, the proportion of RM-only boutons increased, the proportion of UM-only boutons decreased, and that of RM–UM both boutons remained stable (Fig. 2f). To further reveal the dynamic change of bouton representation of RM and UM, we meticulously tracked the activity of the same boutons during the early and late stages (Fig. 2g) and analysed the fate of classified boutons at early stage, and the origin of the classified boutons at the late stage (Fig. 2h and Extended Data Fig. 4). Only around 35% of the boutons maintained their stable representation; most changed their reward selectivity (Extended Data Fig. 4j). For instance, nearly half of the early UM-only boutons became unresponsive, and 20% of them switched to RM-only at late sessions (Fig. 2h). By contrast, once task performance stabilized, RM and UM bouton representations also stabilized (Extended Data Fig. 5a). Notably, in two mice that achieved good performance early, most boutons showed shifts toward more RM boutons with continued training (Extended Data Fig. 5b–e).

Previous studies revealed that the M1 cortical neuron activity pattern was reproducible with learned movement only in the expert mice, whereas similar movements made in early sessions were accompanied by different activity patterns^8,11^. Because the biggest change in bouton representation after learning was the increase of RM-responsive boutons, we tested whether the activity of the RM-responsive boutons was better correlated with movement execution. We analysed the movement trajectories and the activated RM ensembles for each trial pair (Fig. 2i,j). We found a significant relationship between the similarity of movement trajectories and the fraction of activated boutons for each trial pair (Fig. 2k,i; early stage: 1,470 trials, late stage: 2,109 trials, *n* = 13 mice). Notably, such a relationship is only true for late sessions but not early sessions (Fig. 2k,l).

Together, the results indicate that motor learning stabilizes the general relationship between activity and movement in pairs of trials, which is accompanied by changes in the identity of bouton representation of reward outcome.

## Bouton-specific activity and movement behaviour

**Fig. 3:**
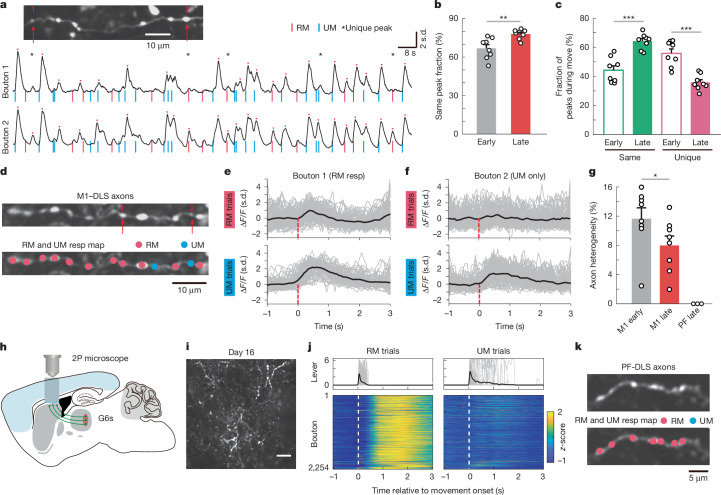
Heterogeneous activity of boutons on the same corticostriatal or thalamostriatal axon. **a**, Top, GCaMP6s image showing a single axon with clear axon and bouton morphology. Bottom, representative Ca^2+^ traces of two boutons on the same axon during RM and UM trials. Red and blue lines represent RM and UM initiation, respectively, red arrowheads show Ca^2+^ transients and stars indicate bouton-specific (heterogeneous) events. **b**, Fraction of unified Ca^2+^ transients at early and late stages of motor learning. *P* = 0.001, two-sided Wilcoxon rank sum test, *n* = 8 mice. **c**, Relative fraction of RM-related same and unique peaks at early and late stages (same: *P* = 3.1 × 10^−4^, unique: *P* = 3.1 × 10^−4^, Wilcoxon rank sum test, *n* = 8 mice). **d**, GCaMP6s image of corticostriatal axon and bouton structures (top), and boutons selective to RM or UM. **e**,**f**, Trial-averaged Ca^2+^ activity from two boutons shown in **d** during RM (top) and UM (bottom) trials on day 14. Both RM- and UM-selective boutons were found on the same axon. Grey represents Ca^2+^ transients in individual trials (Δ*F*/*F*_0_) and black shows average of all trials in one day (day 14). **g**, Axon heterogeneity at M1 early (left), M1 late (middle) and PF late (right) stages (*P* = 0.028, Wilcoxon rank sum test, *n* = 8 mice for M1, *n* = 3 mice for PF). **h**, Schematic of virus injection in PF and imaging in DLS. **i**, Representative image of GCaMP6s-labelled thalamostriatal axons in DLS on day 16. Scale bar, 5 μm. **j**, Top, RM and UM lever individual (grey) and average (black) trajectories. Bottom, corresponding averaged Ca^2+^ activity from 2,254 boutons. **k**, GCaMP6s image of thalamostriatal axons (top) and identified boutons responsive to RM or UM trials (bottom). *P < 0.05, **P < 0.01, ***P < 0.001. Error bars represent s.e.m. Source Data

We next examined whether the bouton-specific activities are related to behaviour outcomes and whether motor learning can further modulate activity patterns of boutons on the same axons. Therefore, we focused the analyses on the populations of boutons on the same axons and aligned their activity with behaviour (Fig. 3a), and consistently, most Ca^2+^ transients were related to movements (RM or UM). By aligning bouton activities between an example pair of boutons, it is clear that although most of the Ca^2+^ transients were present in both boutons, there were ample local activities that only occurred in one bouton but not another (Fig. 3a).

**Extended Data Fig. 6:**
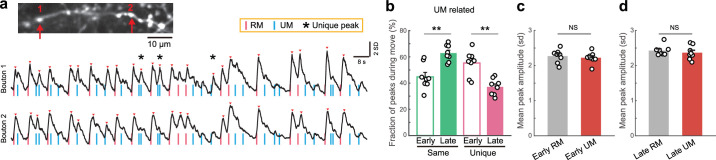
Heterogeneity of UM-related bouton activities located on the same axon. **a**, Top: example of average GCaMP6s image showing a single axon with clear axon and bouton morphology. Bottom, representative Ca^2+^ traces of two distinct boutons (red arrow) located on the same axon. Red vertical line: initiation of RM; blue vertical line, initiation of UM; red arrowhead, detected Ca^2+^ transients; stars, heterogeneous local Ca^2+^ transients. **b**, Relative fraction of UM related same peaks and unique peaks at early and late stage (P(Same)=0.0019, P(Unique)=0.0019, two-sided Wilcoxon rank sum test, n = 8 mice). **c**, Mean amplitude of RM and UM related peaks at early stage of learning (P = 0.33, two-sided Wilcoxon rank sum test, n = 8 mice). **d**, Mean amplitude of RM and UM related peaks at late stage of learning (P = 0.33, two-sided Wilcoxon rank sum test, n = 8 mice). **P < 0.01, NS, not significant. Error bars represent SEM. Source Data

To prevent bias in bouton activities influenced by the highest or the lowest amount of Ca^2+^ transients seen in individual boutons, we first applied detection criteria to define the Ca^2+^ peaks for each bouton. We then compared the timing of the Ca^2+^ peaks between every pair of boutons, categorizing them as either same peaks (Ca^2+^ transients detected in both boutons) or unique peaks (Ca^2+^ transients detected only in one of the boutons). Analysing each entire imaging segment (approximately 4 min) during both the early and late training periods, we observed that about 65% of Ca^2+^ transients were uniformly detected in pairs of boutons (same peaks) during the early training period. Interestingly, the percentage of the same peaks increased to around 80% in late training sessions (Fig. 3b). These data indicated that different boutons on the same axons exhibited surprisingly high heterogeneous activity patterns in vivo—nearly 35% in the early phase of the training—and this heterogeneity could be reduced by motor learning. Because axonal bouton activity was selective to reward outcomes (Fig. 2), we focused on Ca^2+^ transients that occurred during the RM trials. When we compared the percentage of the same peaks versus unique peaks associated with RM trials in the early and late phases of training, we observed a significant increase in the fraction of the same peaks and a significant decrease in the fraction of unique peaks (Fig. 3c). A similar result was seen for UM trials (Extended Data Fig. 6a,b). In addition, we found that RM and UM peak amplitudes were not statistically different at both the early and late stages (Extended Data Fig. 6c,d).

**Extended Data Fig. 7:**
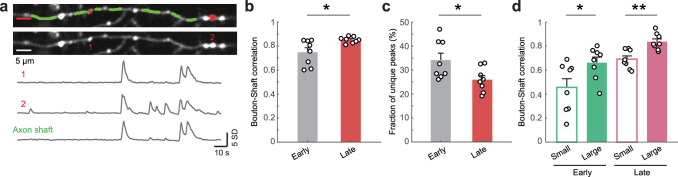
Correlation between axon bouton and shaft activity. **a**, Top: example of average GCaMP6s image showing a single axon with clear axon and bouton morphology. The red circles represent boutons, and the green areas represent axon shaft. Bottom, representative Ca^2+^ traces of two distinct boutons (1 and 2) located on the same axon, and the Ca^2+^ trace of the axonal shaft. **b**, Mean correlation between axon shaft and boutons located on the same axon at early and late stage, note that learning increased the correlation along the same axon (P = 0.015, two-sided Wilcoxon rank sum test, n = 8 mice). **c**, Fraction of unique peaks existed in boutons, but not on axon shaft at early and late stage, learning decreased the heterogeneity between boutons and axon shaft after learning (P = 0.038, two-sided Wilcoxon rank sum test, n = 8 mice). **d**, The correlation for smaller and larger peaks between bouton and axon shaft at early and late stages of learning (P (Early)=0.038, P(Late)=0.003, two-sided Wilcoxon rank sum test, n = 8 mice). * p < 0.05, ** p < 0.01. Error bars represent SEM. Source Data

One of the most notable findings is the existence of individual bouton activities in the absence of Ca^2+^ activity in the axon itself (Supplementary Video 3). To further investigate this, we calculated the mean correlation between shaft calcium activity and individual bouton activity and found that, overall, these were well correlated (Extended Data Fig. 7a,b). Yet, with learning, this correlation was further increased, which is consistent with our finding that bouton responses become more uniform with learning (Fig. 3b). We also further examined the relationship between shaft and bouton activity by calculating the fraction of unique peaks compared with the axonal shaft activity. We consistently found that around 35% of peaks were independent of axonal shaft activity, and learning decreased the fraction of unique peaks (Extended Data Fig. 7c). In addition, we calculated the correlation between bouton and shaft activity for small and large amplitude bouton events, and found that correlation was significantly higher for the large events (Extended Data Fig. 7d), suggesting that isolated bouton events are smaller than bouton events with coinciding shaft activity, both at early and late training stages.

**Extended Data Fig. 8:**
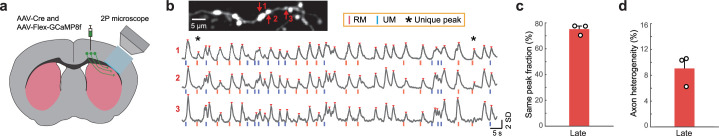
Analysis of axon heterogeneity using GCaMP8f activity. **a**, Schematic diagram showing the sites of virus injection (M1) and imaging (dorsolateral striatum, DLS). **b**, Top: example of averaged GCaMP8f image showing a single axon with clear axon and bouton morphology. Bottom, representative Ca^2+^ traces of three distinct boutons (red arrows) located on the same axon. Red vertical line: initiation of RM; blue vertical line, initiation of UM; red arrowhead, detected Ca^2+^ transients; stars, heterogeneous local Ca^2+^ transients. **c**, fractions of unified Ca^2+^ transients at late stages of motor learning (n = 3 mice). **d**, Axon heterogeneity at late stage of learning (n = 3 mice). Error bars represent SEM. Source Data

**Extended Data Fig. 9:**
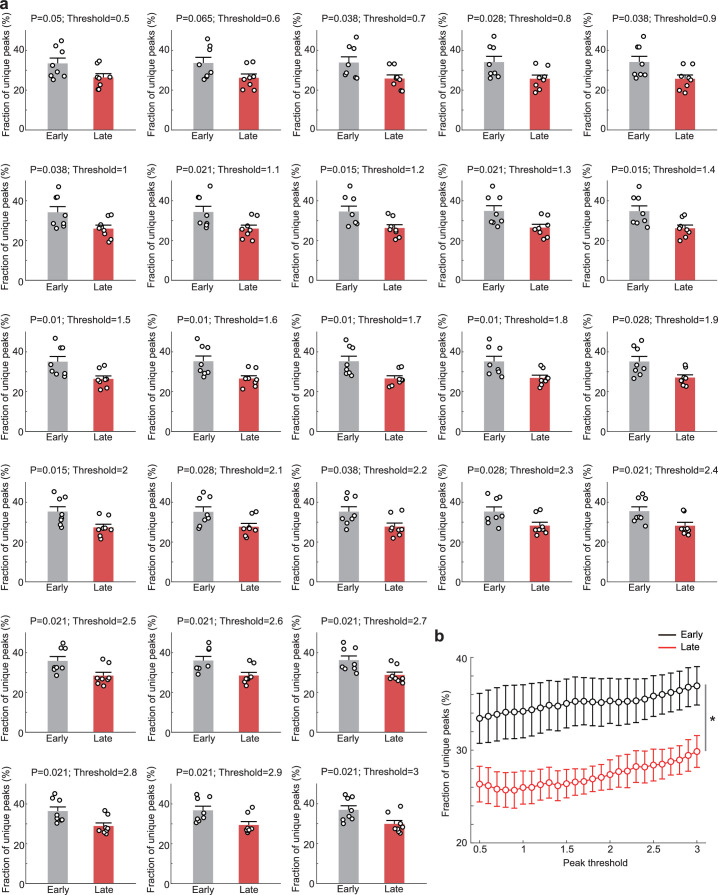
Fraction of boutons Ca^2+^ events absent from axonal shaft across different detection thresholds. **a**, Fraction of unique peaks in boutons (compared to axon shaft) calculated using different peak detection threshold (from 0.5 SD to 3 SD, two-sided Wilcoxon rank sum test, n = 8 mice). **b**, Fraction of unique peaks in boutons plotted against different peak detection thresholds. Note the heterogeneity was not affected by the threshold both at early and late stage, but the fraction of unique peaks at late stage were significantly lower than that at early stage (Early stage, P = 1, one-way ANOVA, n = 8 mice; Late stage, P = 0.96, one-way ANOVA, P = 0.03, two-way ANOVA, between early and late stage, n = 8 mice). *p < 0.05. Error bars represent SEM. Source Data

To rule out GCaMP6s sensitivity limitations, we replicated the key findings using the latest GCaMP8f, which offers higher sensitivity and faster kinetics (Extended Data Fig. 8). We found that an average axon heterogeneity of around 8% in well-trained (late) mice, similar to our previous finding using GCaMP6s (Fig. 3g). We also quantified the fraction of unique peaks across different thresholds for event detection, and consistently found that around 35% of unique peaks, and motor learning decreased the fraction (Extended Data Fig. 9).

**Extended Data Fig. 10:**
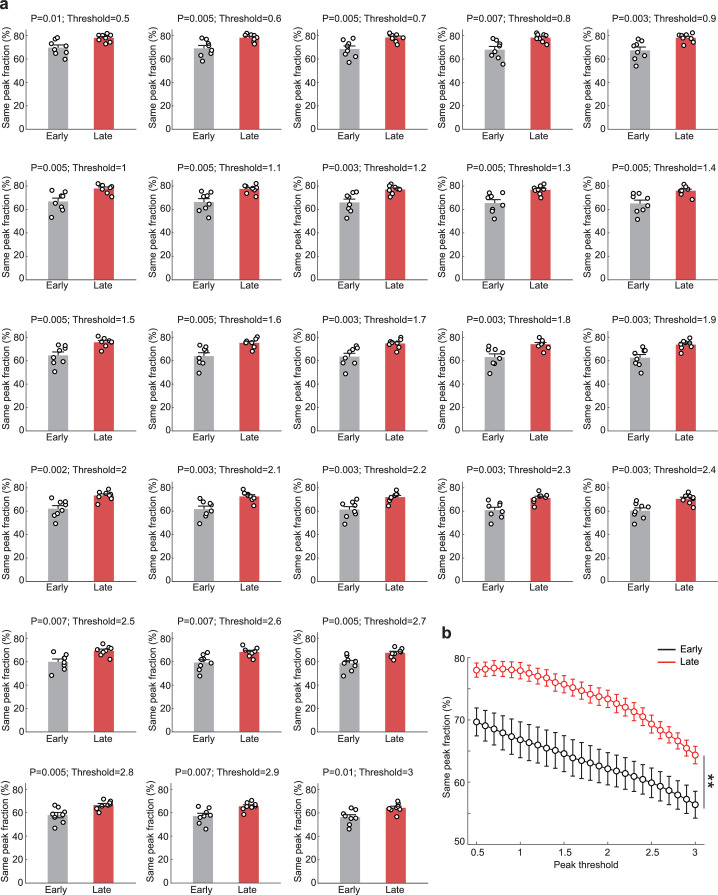
Same peak fraction across different detection thresholds. **a**, Fraction of same peaks between boutons within the same axon calculated using different peak detection threshold (from 0.5 SD to 3 SD, two-sided Wilcoxon rank sum test, n = 8 mice). **b**, Fraction of same peaks between boutons within the same axon plotted against different peak detection thresholds (from 0.5 SD to 3 SD, P = 0.006, between early and late stage, P = 1.9 × 10^−7^, between different thresholds, two-way ANOVA, n = 8 mice). **P < 0.01. Error bars represent SEM. Source Data

**Extended Data Fig. 11:**
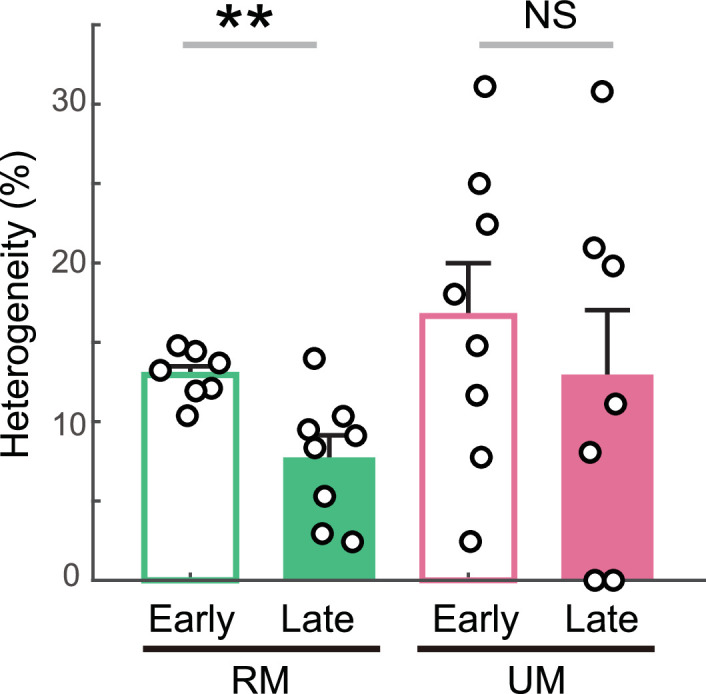
Axon heterogeneity of RM and UM axons. Axon heterogeneity of RM and UM axons at early and late stage (P(RM) = 0.0059, P(UM) = 0.44, two-sided Wilcoxon rank sum test, n = 8 mice). ** p < 0.01, NS, not significant, error bars represent SEM. Source Data

**Extended Data Fig. 12:**
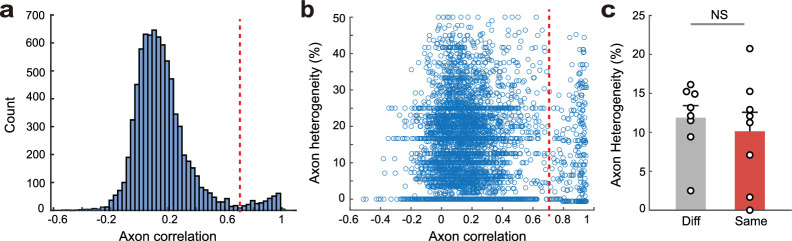
Prediction of axon origin using pairwise correlation in axon activity. **a**, Distribution of pairwise axon correlation (n = 8 mice, 7988 axon pairs). The red dashed line indicates the boundary between two distribution clusters (with axon correlation of 0.7, as the putative cutoff between axon pairs from the same neurons and pairs from different neurons). **b**, Axon heterogeneity plotted against pairwise axon correlation. **c**, Axon heterogeneity in axons originating from putative different and the same neurons (P = 0.5, two-sided Wilcoxon rank sum test, n = 8 mice). NS, not significant. Error bars represent SEM. Source Data

The presence of unique peaks among bouton pairs also raised the question of whether boutons on the same axons could be exclusively RM- or UM-responsive. To address this, we identified the Ca^2+^ transients with RM or UM and mapped their locations along the same axons (Fig. 3d). Of note, within the same axon, boutons predominantly displayed uniform RM or UM selectivity. However, even an RM-dominating axon contained some UM-selective boutons (Fig. 3e,f), and vice versa, a UM-dominating axon also contains RM-selective boutons. In addition, as we showed earlier (Fig. 2h), at the population level, individual axonal bouton RM or UM selectivity could change throughout motor learning. This is also true for boutons on the same axons; the axon heterogeneity (defined by the percentage of RM or UM boutons throughout the axonal segment) decreased after motor learning (Fig. 3g and Supplementary Video 4). These results were robust across various detection thresholds (Extended Data Fig. 10). Further, we analysed the axon heterogeneity for RM axons and UM axons (axons that as a whole have either RM or UM specificity) at early and late stages and found that learning reduced the heterogeneity predominantly within the RM axons (Extended Data Fig. 11). To determine whether axons originate from distinct neurons or if multiple axons stem from the same neuron, we plotted the distribution of pairwise correlations in axon activity (Extended Data Fig. 12a). This analysis revealed two clusters: highly correlated axon pairs (correlation >0.7) and less correlated axon pairs (correlation <0.7). Approximately 4% of total axonal pairs exhibited high correlation, suggesting that they were likely from the same neurons. Conversely, axon pairs with low correlation were presumed to originate from different neurons. To examine how axon origin influences axon heterogeneity, we plotted axonal heterogeneity against axon activity correlation (Extended Data Fig. 12b,c). Axon heterogeneity did not correlate with activity similarity (*r* = −0.06), suggesting bouton heterogeneity is independent of cell of origin.

Besides cortical inputs, the DLS also receives glutamatergic projections from the parafascicular nucleus (PF) in the thalamus^31^. To investigate whether the heterogeneous activities are unique to M1 corticostriatal axons or universal to all glutamatergic projections in DLS, we performed similar longitudinal imaging experiments. In this set of experiments, we injected AAV-GCaMP6s in the PF of the thalamus (Fig. 3h) and imaged PF thalamostriatal axons and boutons in the DLS through the chronic window while simultaneously monitoring the mouse’s behaviour (Fig. 3i and Supplementary Video 5). In contrast to M1 axons, the activities of thalamostriatal axons and boutons did not tile the entire duration of the movement; instead, the activities of thalamostriatal boutons were noticeably more homogenous. Thalamostriatal axons were active almost exclusively during RM trials and showed little activity in UM trials (Fig. 3j). In addition, thalamostriatal axonal boutons did not display heterogeneity in activities along the same axon (Fig. 3g,k).

Together, these findings reveal that boutons on the same M1 axon can exhibit distinct, locally regulated activity patterns that are refined by learning. Furthermore, this heterogeneity is specific to M1 corticostriatal axons. Thalamostriatal axons projecting to the same region in the DLS show a more uniform functional profile compared with corticostriatal axons.

## Structural plasticity of axonal boutons

**Fig. 4:**
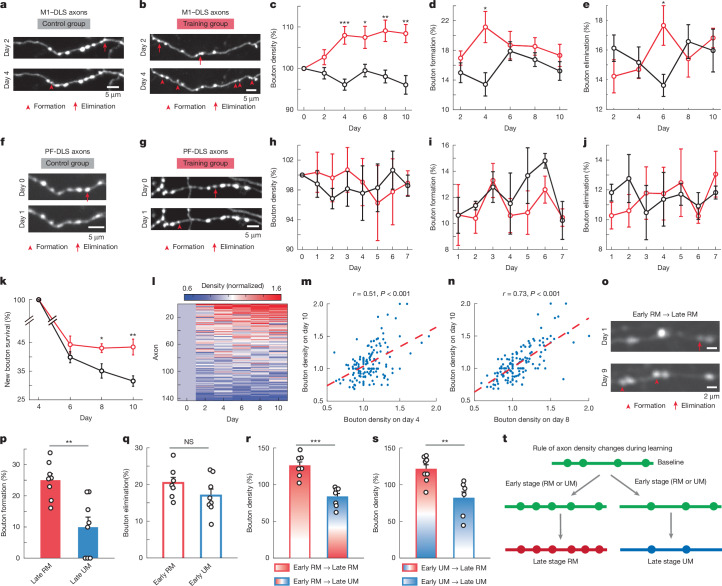
Structural plasticity of corticostriatal and thalamostriatal axonal boutons. **a**,**b**, Repeated imaging shows bouton formation (arrowhead) and elimination (arrow) in control (**a**) and trained (**b**) mice. **c**, Corticostriatal bouton density increased significantly in trained mice versus controls at multiple timepoints (*P* < 0.05; *n* = 143–146 axons from 8–9 mice). Wilcoxon rank sum test, control: *n* = 146 axons, 9 mice; trained: *n* = 143 axons, 8 mice. **d**,**e**, Trained mice exhibited increased bouton formation on day 4 and elimination on day 6. Formation, day 4: *P* = 0.01; elimination, day 6: *P* = 0.027, Wilcoxon rank sum test; control: *n* = 9 mice; trained: *n* = 8 mice. **f**,**g**, Similar imaging of thalamostriatal axons in control (**f**) and trained (**g**) mice showed bouton turnover. **h**, Thalamostriatal bouton density remained unchanged across 7 days (*P* > 0.05, Wilcoxon rank sum test, control: *n* = 46 axons, 3 mice; trained: *n* = 59 axons, 4 mice. **i**,**j**, No significant differences in bouton formation (**i**) or elimination (**j**) in thalamostriatal axons between groups. *P* > 0.05 for days 1–7, Wilcoxon rank sum test; control: *n* = 3; trained: *n* = 4 mice. **k**, New boutons formed on day 4 and survived. Day 8: *P* = 0.027, day 10: *P* = 0.0037, two-sided Wilcoxon rank sum test; control: *n* = 9 mice; trained: *n* = 8 mice). **l**–**n**, Bouton density at earlier stages correlated with later density (**l**) (day 10 versus day 4 (**m**): *r* = 0.51, *P* = 7.46 × 10^−11^; versus day 8 (**n**): *r* = 0.73, *P* = 9.23 × 10^−25^; Pearson’s correlation, *n* = 143 axons). **o**, GCaMP6s images of averaged GCaMP6s signal from day 1 and 9 reveal bouton formation and elimination. **p**,**q**, RM boutons formed at a higher rate than UM boutons at the late stage (*P* = 0.0019), with no difference in elimination rates. **r**,**s**, Bouton density increased in early RM-responsive axons (**r**; *P* = 5.8 × 10^−4^) and decreased in early UM-responsive axons (**s**; *P* = 0.0047, Wilcoxon rank sum test, *n* = 8 mice). **t**, Model for bouton turnover on axons with motor learning. Bouton density increases in axons that become RM-responsive during learning and decreases in those that become UM-responsive. *P < 0.05, **P < 0.01, ***P < 0.001; NS, not significant. Error bars represent s.e.m. Source Data

The changes in activity patterns and reward representations of M1 corticostriatal axonal boutons indicate a dynamic regulation of corticostriatal synaptic transmission. In postsynaptic striatal SPNs, dendritic spines—where the glutamatergic corticostriatal synapses are formed^32,33^—undergo significant activity-dependent structural changes, for example, in mouse models of movement disorders^17–19^. Here, we explore whether motor learning could result in dynamic remodelling of presynaptic axonal bouton structures. Using in vivo two-photon imaging, we tracked individual corticostriatal and thalamostriatal axons labelled with eGFP across 11 days. In the training group, the mice were trained on the cued lever-pushing task starting on day 1, and the control group underwent identical procedures, including water restriction, habituation and water consumption from the licking port, but without training to push the lever. By comparing images taken from two timepoints, we identified axonal boutons as newly formed, eliminated or stable (Fig. 4a,b). We calculated total bouton numbers for each axon to assess whether bouton density changes following motor learning. We found that the corticostriatal axonal bouton density was significantly increased from day 4 and persisted throughout the training period (control: *n* = 146 axons, *n* = 9 mice; training: *n* = 143 axons, *n* = 8 mice; Fig. 4c). To further understand the process of motor learning-induced structural plasticity, we quantified the rate of newly formed and eliminated M1 axonal boutons. Motor learning induced a transient increase in the formation of boutons on day 4 (Fig. 4d), accompanied by enhanced bouton elimination on day 6 (Fig. 4e). Using the same approach, we also analysed thalamostriatal axonal boutons (Fig. 4f,g). By contrast, thalamostriatal bouton density did not show statistically different changes throughout the training days (control: *n* = 46 axons, *n* = 3 mice; training: *n* = 59 axons, *n* = 4 mice; Fig. 4h). In addition, even though thalamostriatal axonal boutons still showed ongoing turnonver, motor learning did not induce similar transient increase in either formation or elimination as corticostriatal boutons (Fig. 4i,j).

**Extended Data Fig. 13:**
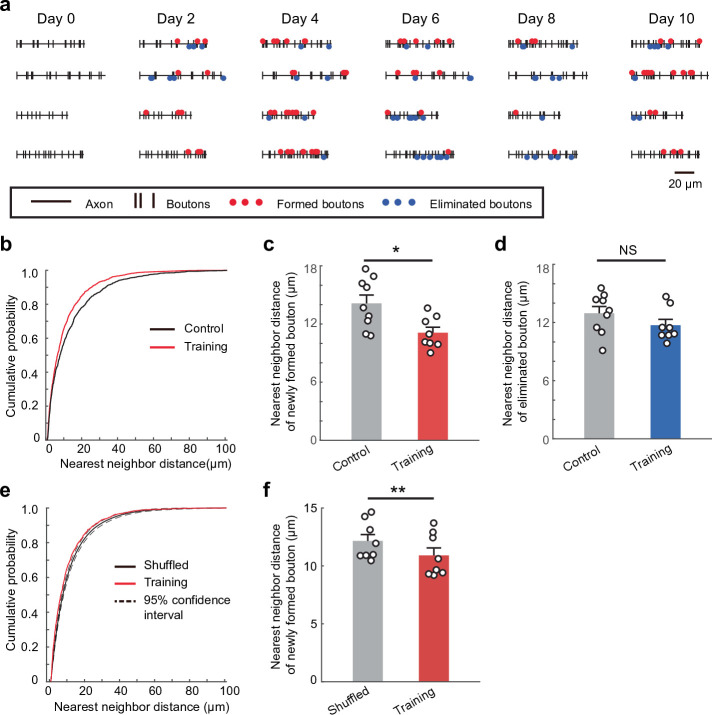
Spatial distribution of newly formed and eliminated boutons. **a**, Spatial distribution of newly-formed and eliminated boutons along M1 axons throughout training. Black vertical lines indicate boutons that persisted throughout the imaging sessions, red circles indicate newly formed boutons, and blue circles indicate eliminated boutons. **b**, Cumulative distribution of nearest neighbor distance for newly formed boutons in the control (black) and training (red) group (P = 2.47 × 10^−6^, two-sided Kolmogorov–Smirnov test). **c**, Average nearest neighbor distance of newly formed boutons in control (black) and training group (red) mice (P = 0.01, two-sided Wilcoxon rank sum test, control: n = 9 mice; training: n = 8 mice). d, Average nearest neighbor distance of eliminated boutons in control (black) and trained (red) mice (P = 0.17, two-sided Wilcoxon rank sum test, control: n = 9 mice; training: n = 8 mice). **e**, Cumulative distribution of nearest neighbor distance for newly formed boutons in shuffled (black) and training (red) group. **f**, Average nearest neighbor distance of newly formed boutons in shuffled (black) and training group (red) mice (P = 0.0014, two-sided paired t-Test, n = 8 mice). *p < 0.05, **p < 0.01, NS, not significant. Error bars represent SEM. Source Data

Previous studies showed that newly formed dendritic spines in M1 layer five pyramidal neurons are preferentially stabilized during training^1,34^. To test whether motor learning stabilizes newly formed boutons, we analysed their fate. In trained mice, newly formed axonal boutons were more likely to persist; in controls, most were eliminated (Fig. 4k). Previous work showed that motor learning led to a selective strengthening of M1 motor engram neuron outputs formed onto clustered spines of postsynaptic striatal SPN dendrites^6^. Therefore, we examined the position of newly formed and eliminated boutons along each axon (Extended Data Fig. 13a) and plotted the cumulative distribution of nearest neighbour distance (NND) for newly formed boutons in the control and training groups. We found that the bouton pairs were significantly closer in space in trained mice compared with the control mice (Extended Data Fig. 13b). The average NND of newly formed boutons was significantly shorter in trained mice compared with control mice (Extended Data Fig. 13c). However, the average NND of eliminated boutons was not different between groups, suggesting that eliminated boutons were not clustered along the axons (Extended Data Fig. 13d). By comparing the cumulative NND distribution of newly formed boutons with that of shuffled boutons, we observed a leftward shift in the cumulative NND distribution for the training group. Furthermore, a portion of the cumulative distribution curve exceeded the upper 95% confidence interval of the shuffled bouton distribution, indicating significant clustering within this range of NNDs (approximately 5–30 µm). Together, these analyses suggest the reduced NND following training is not simply caused by a higher bouton density, but that there is indeed increased clustering of corticostriatal boutons after motor learning.

**Extended Data Fig. 14:**
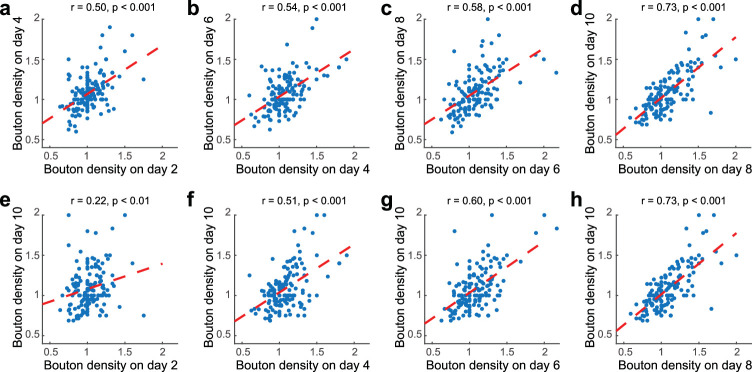
Changes of bouton densities across days. **a-d**, The normalized bouton density plotted against the density on adjacent imaging days. **e-h**, Bouton density on day 10 plotted against its density on day 2 (**e**), day 4 (**f**), day 6 (**g**), and day 8 (**h**). The bouton densities across days were positively correlated (P = 2.29 × 10^−10^ (**a**), P = 3.64 × 10^−12^ (**b**), P = 5.28 × 10^−14^ (**c**), P = 9.23 × 10^−25^ (**d**), P = 0.0082 (**e**), P = 7.46 × 10^−11^ (**f**), P = 3.72 × 10^−15^ (**g**), P = 9.23 × 10^−25^ (**h**), two-sided Pearson’s correlation, n = 143 axonal segments). Source Data

The newly formed boutons form clusters along the axons, but the eliminated boutons did not have a similar spatial arrangement, indicating that bouton structural remodelling may be axon-specific. Therefore, we plotted bouton density changes throughout the learning process and sorted the axons on the basis of their maximum density change (Fig. 4l). Even though the average bouton density calculated based on all axons increased during motor learning (Fig. 4c), the change in axon density diverged into two groups: axons exhibiting increased density at the early stage tended to persistently increase their density throughout the late stage, whereas axons decreased their density at early stage of training tended to remain lower density at the late stage (Fig. 4l). When we plotted the density of each axon on day 10 against those of day 4 and day 8, it revealed a significant positive linear correlation (Fig. 4m,n and Extended Data Fig. 14).

Together, these data suggest that the early stages of axonal bouton development influence final bouton density. It is possible that axons engaged in the early stages are more likely to continue to transmit corticostriatal synaptic information, and learning can further strengthen connectivity and increase synaptic transmission efficacy. Our results demonstrated that learning could change the axonal bouton selectivity to movement based on reward outcome; in particular, learning increased the proportion of RM-related boutons but reduced the UM-related ones (Fig. 2f). This raised intriguing questions: such as whether the newly formed or eliminated axonal boutons are activity-dependent; and if so, whether they are dependent on the reward outcome. To address this, we used the calcium imaging dataset, in which high-resolution averaged GCaMP6s images obtained from the same axons in both early- and late-stage imaging sessions could be used to clearly identify newly formed and eliminated boutons (Fig. 4o) and examined their activity pattern in relation to RM or UM. Of note, when we calculated the bouton formation rate in RM-related versus UM-related axons identified at late stage, we observed a significantly higher bouton formation rate in RM axons compared with UM axons (256 RM axons, 46 UM axons, *n* = 8 mice; Fig. 4p). However, the bouton elimination rates were similar (215 RM axons and 95 UM axons identified at early stage of learning, *n* = 8 mice, Fig. 4q). Next, we analysed the bouton density changes for those functionally identified axons. We found that if axons were identified as RM-related at the early stage and maintained their identity, the bouton density was higher compared with those that were identified as RM-related at the early stage and became UM-related at the late stage (Fig. 4r). Conversely, axons identified as UM-related at the early stage would have a higher bouton density if they became RM-related compared with those that remained UM-selective (Fig. 4s). Together, these data suggest that the formation, elimination and maintenance of the newly formed boutons and the overall bouton density of the axons are associated with the activity of axonal boutons and dependent on behavioural outcomes (Fig. 4t).

## Discussion

This study reveals how motor learning reshapes corticostriatal circuits at the level of individual axonal boutons. By combining two-photon imaging with a cued lever-pushing task, we show that corticostriatal bouton activity is movement-related, modulated by reward and structurally remodelled during learning. Previous studies using somatic Ca^2+^ imaging or in vivo recordings in the primary motor cortex revealed the formation of movement-specific cortical ensembles, whose firing covers the motion sequence and the increases in activity correlation after motor skill learning^11,35^. Our results extend previous findings on M1 somatic dynamics by demonstrating that bouton populations also develop stable, reproducible patterns that are aligned to movement sequences (Fig. 1i–l). In addition, bouton activity distinguishes rewarded trials from unrewarded trials, and this selectivity sharpens with learning (Fig. 2a–c). By closely examining the activities of different boutons formed on the same axon, we found surprisingly heterogeneous activity patterns on different boutons even though they are only a few micrometres away on the same axon (Fig. 1m,n). Furthermore, these unique local heterogeneous responses are shaped by motor learning in several ways. First, motor learning can enhance the consistency of the activity responses across boutons on the same axon (Fig. 3a–c). Second, the bouton RM or UM selectivity becomes more uniform at late phases (Fig. 3g and Extended Data Fig. 6). Finally, axon boutons undergo activity-dependent structural plasticity (Fig. 4). Importantly, all these plasticity events occur specifically on corticostriatal axons, but not on the thalamostriatal boutons projecting to the same region. The lack of heterogeneity and structural plasticity at thalamostriatal axonal boutons may be explained by their different functional activity profile. Unlike M1 corticostriatal axons, the activities of thalamostriatal axons and boutons were homogenous, being active almost exclusively during RM trials (Fig. 3j). PF thalamostriatal axons may be encoding salient environmental cues rather than movements^36^. Similarly to M1 cortical ensembles, a previous study showed that dopamine D1 and D2 SPNs exhibited sequential firing spanning over the entire movements, with D1 SPNs activated predominantly during the lever movement period and D2 neurons activated predominantly after the lever-pushing movement^16^. Overall, the corticostriatal bouton activity is similar to the combined activity patterns of D1 and D2 SPNs, consisting of movement and post-movement-related activities. This aligns with the fact that both D1 and D2 SPNs receive inputs from M1 neurons, and their activities are driven by glutamatergic inputs^15^. However, our results suggest that there may be a preferential connection between boutons with activity earlier during the movement to D1 SPNs and boutons with activity after movement to D2 SPNs, which could be addressed in future studies—for example, by simultaneous imaging and/or recording of presynaptic M1 axons and postsynaptic SPNs. Corticostriatal axon and bouton activities are refined throughout motor learning at both the population and single axon or bouton levels. Overall, our longitudinal results provided a link between subcellular synaptic and system-level dynamics to reveal how corticostriatal ensembles are formed and maintained throughout learning.

An unexpected finding is the markedly heterogeneous activity patterns among nearby boutons formed on the same axon. Decades of neuroscience research have yielded a classic model of how axons convey neuronal output to downstream postsynaptic targets^37,38^. Because of the high expression levels of voltage-gated Na^+^ and K^+^ channels^39,40^, forward propagation of action potentials is generally considered highly reliable and functions as a digital signal (all or none), which ensures faithful outputs^28–30,40^. In certain specialized synapses in sensory receptor cells, analogue graded potential is used to increase the fidelity and capacity of synaptic information, including the rod bipolar–AII amacrine cell ribbon synapse in the retina^41^ and the hair cell ribbon synapse in the inner ear^42–44^. A combination of analogue and digital coding of axonal transmission also exists—for example, at hippocampal mossy fibres, transient subthreshold depolarizations can modulate action potential-evoked transmitter release^45^ by altering the waveform of the action potential (for example, amplitude and duration)^46^. However, the heterogeneous responsive pattern revealed here represents an additional novel mechanism for information transmission at corticostriatal output. The en passant axonal boutons can function as a demultiplexing processer, where postsynaptic targets can receive distinct patterns of axonal output even though these targets are innervated by the same axon.

Furthermore, we demonstrate that distinct patterns of axonal bouton activity are behaviourally relevant, and motor learning can significantly increase the uniformity of bouton activity along the same corticostriatal axon (Fig. 3). One hallmark of motor learning is the formation of stereotypic movement patterns^8,11,16,35^. In addition, motor learning reduces motion variation and jitter^8,11,16,35^. On the population level, the increased activity correlation across motor cortical neurons^11,35^, striatal neurons^16^, and here, corticostriatal axons and boutons, and the formation of stable ensembles make the corticostriatal circuits more efficient in encoding and driving movement. On the single-axon level, the mechanism that we uncover here can also contribute to increased efficiency, where axonal boutons become more uniform in activity patterns and RM or UM selectivity through activity-dependent axonal plasticity. Mechanistically, what contributes to the generation of different bouton activity patterns remains unknown. This parallel but distinct output might be due to differential inputs via axon–axonic synapses. Recent studies have shown that local axonal excitatory postsynaptic potentials could be evoked at dopaminergic axonal terminals in the striatum by activation of nicotinic acetylcholine receptors^47,48^, and conversely, activation of type A GABA (γ-aminobutyric acid) receptors (GABA_A_ receptors) could also locally dampen axonal spikes and action potential-evoked dopamine release^49^. In addition to nicotinic acetylcholine receptors and GABA_A_ receptors, corticostriatal axons also express receptors of various neuromodulators, such as dopamine D1 and D2 receptors^50^. It is possible that these ionotropic and metabotropic receptors contribute to the local modulation of axonal bouton activity patterns.

## Methods

### Animals

All experiments were performed in accordance with protocols approved by the Stanford University Animal Care and Use Committee, in accordance with the National Institutes of Health’s Guide for the Care and Use of Laboratory Animals. All mice were maintained with a 12 h:12 h light:dark cycle at a room temperature of 22 °C with humidity control (30–70%). Both male and female wild-type mice (C57BL/6 J, aged 7 weeks to 6 months) (Jackson Laboratory) were used.

### Surgical procedures

We performed surgeries on mice under isoflurane anaesthesia (1.5% in 0.5 l min^−1^ O_2_). We used a combination of Cre and FLEX-GCaMP6s, FLEX-GCaMP8f or FLEX-eGFP viruses to achieve sparse labelling. To drive the expression of GCaMP6s or GCaMP8f in the motor cortex, we stereotaxically injected a mixture of AAV1-CAG-FLEX-GCaMP6s (100842-AAV1, 1:1) or AAV9-syn-FLEX-jGCaMP8f (162379-AAV9) and AAV5-hSyn-Cre (105553-AAV5, 1:200 diluted in saline) into the caudal forelimb area of the motor cortex (from bregma, anteroposterior (AP): 0.3 mm, mediolateral (ML): 1.5 mm; and from dura, dorsoventral (DV): −0.7 mm). Similarly, for structural imaging, we injected a mixture of AAV5-CAG-FLEX-eGFP (51502-AAV5, 1:1) and AAV5-hSyn-Cre (105553-AAV5, 1:1,000 diluted in saline). For expression of GCaMP6s in the thalamus, we injected a mixture of AAV1-CAG-FLEX- CaMP6s (100842-AAV1, 1:1) and AAV5-hSyn-Cre (105553-AAV5, 1:200 diluted in saline) into the PF (from bregma, AP: −2.3 mm, ML: 0.63 mm; and from dura, DV: −3.25 mm). A total volume of 100–300 nl was injected over 10 min, using a micro pump (WPI). To prevent viral backflow, the pipette was left in situ in the brain for 15 min post-injection before withdrawal. Upon completion of the procedure, the incision site was sutured, and the mice were returned to their home cage once they recovered from anaesthesia.

For the implantation of the chronic imaging window, 3–30 days after virus injection, we anaesthetized the mice with isoflurane (1.5% in 0.5 l min^−1^ O_2_). Following scalp removal, a titanium head plate was affixed firmly to the skull using super glue and dental cement (Lang Dental). A circular craniotomy with a diameter of approximately 2.4 mm was performed above the dorsal lateral striatum, centred at the coordinates (AP: 0.3 mm, ML: 4.0 mm). We aspirated the cortical tissue above the striatum using a 27-gauge needle at a 30° angle towards the surface of the corpus callosum^16,51^. Subsequently, a cannula was inserted above DLS. The cannula consisted of a stainless-steel tube (~2.4 mm diameter, ~1.6 mm length) and a 2.4 mm round coverslip attached to one end of the tube using adhesive (Norland optical adhesive)^16,51^. We then used Kwik-Sil and dental cement to fix the cannula and cover the exposed skull. Mice were returned to their home cage after they recovered from anaesthesia.

### Two-photon imaging

In vivo imaging experiments were conducted using a commercial two-photon microscope (Bergamo II, Thorlabs), operated with ThorImage software. We used a 16×/0.8 NA objective (NIKON), covering a field of view (FOV) size ranged from 120 × 120 to 200 × 200 µm (1,024 × 1,024 pixels). A mode-locked tunable ultrafast laser provided 925 nm excitation for two-photon imaging (Insight X3 Spectra-physics). For calcium imaging, we imaged awake mice when they were performing the lever-pushing task. Imaging data were synchronized and recorded with a PCIe-6321 card (National Instrument) to capture image frame-out timing and behavioural events, encompassing cue, rewards, punishments, licking behaviour, and lever displacement. Time-lapse movies were acquired at an approximate frame rate of ~15 Hz. One to three days were imaged for the early stage and one to six days were imaged for the late stage. For imaging the same population of axons and boutons, same FOVs were imaged between early and late stage. The first 3 days were defined as the early stage, late stage was the days when mice learned the task (≥8 days). For example, one mouse was imaged on days 1–3 and days 9–11, then day 1–3 were defined as early stage, and days 9–11 were defined as the late stage. For corticostriatal axons using GCaMP6s, 13 mice were used in functional calcium imaging, including 8 mice imaged the same axons and boutons at the early and late stage, another 5 mice imaged different FOVs at the early and late stage of learning. For thalamostriatal axons using GCaMP6s, three mice were imaged at late stage of learning. Another three mice were imaged using GCaMP8f.

For structural imaging, mice were anaesthetized with 1–1.5% isoflurane and a heating pad was used to keep normothermia. Image stacks were acquired via real-time averaging of 20 frames, with a *z*-step of 1 μm to ensure precise axial resolution. For corticostriatal axons, 2–4 regions of interest (ROIs) were imaged per mouse, and these ROIs were repeatedly imaged every other day. Eight mice were used in structural imaging for the training group, and nine mice were used for the control group. For thalamostriatal axons, ROIs were imaged daily, three mice were used for the control group and four mice were used for the training group.

### Cued lever-pushing task

The cued lever-pushing task was conducted as previously described^16^. In brief, mice were subjected to water restriction at 1 ml per day for three days. The lever-pushing task training started three days after water restriction and habituation. During habituation, mice were head-fixed and received water from the water tube. After starting the training, mice remained water restricted but received water during the training. Lever displacement was continuously monitored using a potentiometer, converting it into voltage signals, and recorded through a PCIe-6321 card (National Instrument). A custom LabVIEW program governed the training paradigm, precisely controlling cue presentation, reward delivery, punishment, and the determination of lever-pushing threshold crossing. Each trial was initiated with a 500 ms, 6-kHz pure tone as the cue. Mice received a water reward (approximately 8 μl) when they pushed the level surpassed the designated threshold (0.5 mm during the initial training on day 1, later increased to 1.5 mm for subsequent sessions) within the allocated task period. Failure to meet the threshold or absence of lever pushing during the task period resulted in the presentation of white noise. The ITI was either fixed at 4 s or randomly varied between 3 and 6 s. Lever pushing during the ITI incurred an additional timeout equivalent to the ITI duration for that specific trial. The task period was 30 s during the first session and then reduced to 10 s for subsequent sessions. The ITI was defined as the time from the end of the last trial (reward or punishment) to the start of the next trial (cue) and does not include the allocated task period. In a subset of mice, we randomly added reward delay trials and reward omission trials on one imaging day while imaging the same population of boutons. In reward delay trials, the reward was not delivered immediately after the lever exceeded the threshold, but was delivered 1 s after the lever exceeded the threshold. In reward omission trials, the reward was not delivered even when lever exceeded the threshold. In a further subset of mice, we included cue-only or punishment-only trials after mouse finished performing the lever-pushing task. A total of 37 mice were trained, mice learned the task within 3 weeks, including 19 mice for calcium imaging and, 12 mice for structural imaging, and 6 mice used for behaviour training.

### Movement behaviour analysis

To identify movement bouts, we first determined a threshold to separate the resting and movement period. Movement bouts separated by less than 500 ms were considered continuous and were combined together^11,16^. The start time was identified as the point where the lever position crossed a threshold that exceeded the resting period, while the end time was determined by detecting the moment when the lever position fell below the threshold^11,16^. To ensure the integrity of the baseline before each movement, we adopted a specific criterion. If there were any other movements occurring within a 3-s window before a particular movement, the latter was excluded from further analysis. This exclusion step was implemented to guarantee the cleanliness and reliability of the baseline period, thus enhancing the accuracy of subsequent analyses. RM was defined as lever pushes that exceeded the threshold during the task period, while UM was those lever pushes that failed to exceed the threshold during the task period, or lever pushes during ITI.

### Activity pattern correlation and its relationship to movement trajectory correlation

The activity pattern correlation was calculated based on single trial pairs using population bouton activity for each mouse. Therefore, the activity of all responsive boutons in an imaging FOV were concatenated for each trial in the same order and the trial-to-trial correlation of this population activity vector was calculated. Activity pattern correlation and movement trajectory correlation were calculated for each trial pair using MATLAB function corrcoef. For all trial pairs in one day, we used bins −0.2 to 0, 0 to 0.2, 0.2 to 0.4, 0.4 to 0.6, 0.6 to 0.8 and 0.8 to 1 to average all data points based on movement trajectory correlations. Then the activity pattern correlation was plotted against the movement trajectory correlation for each mouse.

### Fraction of activated ensemble difference and its relationship to movement trajectory correlation

Percentage of activated ensemble difference was calculated based on each pair of trials, if *a* is the number of activated bouton ensemble in trial 1, and *b* is the number of activated bouton ensemble in trial 2, then the fraction of activated ensemble difference for this trial pair is defined as $\frac{|a-b|}{0.5\times (a+b)}$, in which |*a* − *b*| was the difference in the number of activated ensembles, and $0.5\times (a+b)$ was the average number of activated ensembles for the trial pair. Then we calculated correlation of the movement trajectory for each trial pair using MATLAB function corrcoef. For all trial pairs in one day, we used bins −0.2 to 0, 0 to 0.2, 0.2 to 0.4, 0.4 to 0.6, 0.6 to 0.8 and 0.8 to 1 to average all data points based on movement trajectory correlations. Then the percentage of activated ensemble difference was plotted against the movement trajectory correlation for each mouse.

### Image processing and analysis

For Ca^2+^ image analysis, lateral motion artifacts were corrected using the ImageJ plugin Turboreg^52^ or the efficient subpixel image registration algorithm^53^. ROIs for axons, axonal shafts and boutons in FOV were manually drawn using Adobe Photoshop session-by-session. For the same FOV imaged both in early and late stages, only boutons with clear bouton morphology that could be identified in all sessions by visual inspection were selected and further analysed. On average 44.3 ± 7.9 (ranging from 10–85) axon segments were analysed per mouse with an average of 10.66 ± 1.66 boutons (early) and 11.2 ± 1.9 boutons (late) per axon segment for M1-DLS projections and 26 ± 16.5 (ranging from 16–45) axon segments per mouse with an average of 6.47 ± 0.88 boutons (late) for PF-DLS projections.

To extract the calcium signals for each axon or bouton, we averaged the fluorescence intensity of all labelled pixels to obtain the raw fluorescence trace. To calculate *F*_0_, we utilized a 30-s sliding window, where the 30th percentile of raw fluorescence within the window was designated as *F*_0_. Δ*F*/*F* was computed as (*F* − *F*_0_)/*F*_0_ for each individual axon and bouton^54^. For data presentation, a *z*-score of this Δ*F*/*F* trace was further calculated.

**Extended Data Fig. 15:**
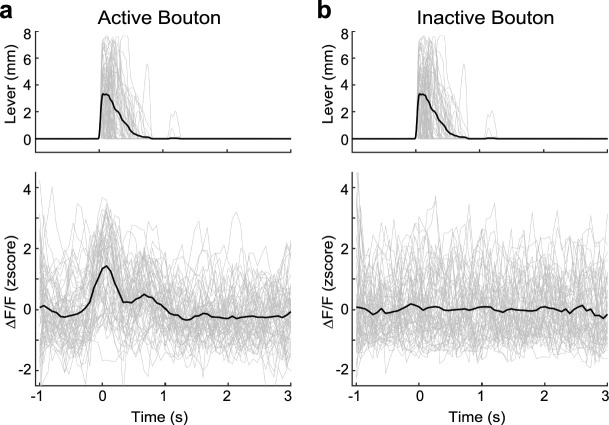
Example traces showing movement trajectories and calcium activity of an active (a) and inactive (b) bouton. Gray traces represent single trial movements or calcium activity, while black traces represent the mean. Source Data

To confirm that the observed signal was not caused by motion artifacts, we plotted the fluorescence signal of inactive boutons and found no detectable activity across many movement trials (Extended Data Fig. 15).

For structural imaging, individual boutons were identified as swellings along thinner axon shafts, and were manually identified, marked, and tracked across multiple imaging sessions using the custom written script (MATLAB). Only high-quality images displaying sparsely labelled axons, with distinct axon and bouton structures, were selected for subsequent quantification. Analysis of bouton dynamics, including formation and elimination, was performed by comparing boutons between two adjacent imaging sessions. Boutons were classified as ‘persistent’ if they were present in both images, determined through their positions relative to nearby boutons within the same axon. An eliminated bouton was the one that appeared in the initial image but not the second image. A newly formed bouton was the one that was absent in the initial image and then appeared in the second image. The bouton survival rate was calculated as the percentage of boutons formed during day 4 of training that remained present in subsequent training sessions (days 6, 8 and 10).

### Identification and classification of RM and UM axon and bouton

The activities of individual axons or boutons in both RM trials and UM trials were aligned to the movement onset, spanning a time window from 1 s before movement initiation (served as the baseline) to 3 s after the movement onset. Subsequently, we calculated the average activity across all trials within this aligned time window. To identify responsive boutons, we examined the peak value of each bouton within the time window (−0.2 to 3 s relative to the movement onset). Boutons were considered responsive if the difference between the peak fluorescence value and the 5th percentile of the averaged activity exceeded 90% of the s.d. For the identification of responsive axons, we plotted histograms of all peak values in RM and UM trials for each mouse. Utilizing a bin size of 0.1× s.d., the peak bin values were determined for both RM and UM distributions, and the threshold was established as the mean of the corresponding peak positions in RM and UM. If the calculated threshold, based on the histogram distribution, exceeded 1× s.d., the final threshold was set at 1× s.d. Responsive axons were identified if the difference surpassed the threshold by comparing each axon’s peak value to the 5th percentile of the averaged activity. Subsequently, axons or boutons were categorized based on their responsiveness in RM and UM trials. Those identified as responsive exclusively in RM trials were classified as RM-only axons or boutons, while those responsive only in UM trials were categorized as UM-only axons or boutons. Axons or boutons showing responsiveness in both RM and UM trials were designated as RM–UM both axons and boutons. To simplify, we combined the RM-only and RM–UM both categories, grouping them as RM, RM-responsive or RM-related axons and boutons. To calculate the delay reward related boutons, we first calculated the activity peak time for each bouton during RM and delay reward trials. If the activity peak time of a bouton was postponed more than 0.93 s, we categorized this bouton as delay reward modulated bouton. Those delay reward modulated boutons were considered to be modulated by reward, rather than movement. To analyse the activity of those reward modulated boutons in reward omission trials, we averaged the calcium activity over a window of 1.67 s to 2.33 s relative to movement onset for delay reward trials and 0.67 s to 1.33 s relative to movement onset for omission trials.

### Ca^2+^ event detection and identification of same or unique peaks

To detect Ca^2+^ events, we employed the Matlab findpeaks function with the following criterion^55^: *z*-scored Δ*F*/*F*_0_ exceeding 1× s.d. To compare events between pairs of boutons, we considered any events occurring within 670 ms of each other as ‘matched’ and defined them as the same peak^56^, while those peaks that cannot find matched peaks were defined as unique peaks. If the same peaks or unique peaks occurred during a time window 330 ms before and 670 ms after the onset of RM or UM, those peaks were classified as RM or UM-related same or unique peaks, respectively. To calculate the same or unique peak fraction, we divided the number of same peaks with total peaks based on each bouton pair or bouton–shaft pair, and averaged the results over all boutons within one axon, then averaged over all axons in one mouse.

### Principal components analysis

We used PCA to project each trial into a lower-dimensional space to discern the low-dimensional embedding of individual boutons during RM and UM trials. Initially, the activity of each bouton was averaged across all RM or UM trials, and the averaged activities were then concatenated for each bouton. We recorded the results in a data matrix where each column represented the concatenated trial-averaged RM and UM activity of one bouton. The size of the matrix was 2*M* × *N*, with *M* denoting the number of timepoints per RM or UM trial (ranging from –1 to 3 s relative to movement onset), and *N* representing the number of boutons. Subsequently, PCA was conducted across the timepoints of concatenated RM and UM trials, capturing the first three principal components to represent the RM and UM trials in a visually informative 3D principal component space. Each bouton was depicted as a distinct dot within this space, facilitating clear visualization and discrimination of the bouton responses during both RM and UM trials. We used the Matlab pca function to perform dimension reduction.

### PCA trajectory and calculation of selectivity index

PCA was conducted using the Matlab pca function on each continuous imaged segment (4,000 frames by *n* boutons, frame rate: 15 Hz), utilizing the first three principal components to represent the ensemble activity of boutons. Then we aligned the first three principal components from 1 s before to 3 s after each RM and UM onset to generate single RM or UM neural trajectories in the PCA space. We used activity trajectory selectivity index to measure the selectivity of bouton activity towards RM or UM, a method modified from a previously published paper^57^. The activity trajectory selectivity index for an RM trial was defined as (*d*_to mean UM trajectory_ – *d*_to mean RM trajectory_)/(*d*_to mean RM trajectory_ + *d*_to mean UM trajectory_), where *d*_to mean UM trajectory_ is the Euclidean distance between the single RM trial trajectory and the mean UM (RM) trajectory, which was computed frame by frame. The mean RM and UM trajectories were the averages of all RM and UM trajectories, respectively. For example, if the first three principal components of the first frame of a RM trial are $(a,b,c)$, while the first three principal components of the first frame of the mean UM trial are $(x,y,z)$, then the *d*_to mean UM trajectory_ is $\sqrt{{(a-x)}^{2}+{(b-y)}^{2}+{(c-z)}^{2}}$. Similarly, the activity trajectory selectivity index for a UM trial was defined based on distances as (*d*_to mean RM trajectory_ – *d*_to mean UM trajectory_)/(*d*_to mean RM trajectory_ + *d*_to mean UM trajectory_). The trajectory selectivity index essentially measures how closely individual trajectories match the mean trajectories of their respective trial type versus the opposite type. For example, for an RM trial, an index score of 1 means the single trial trajectory was at the same point in PCA space as the mean RM trajectory, and an index score of −1 means the single trial trajectory was at the same point in state space as the mean UM trajectory.

### Axon–axon and bouton–shaft correlation analysis

Axon–axon and bouton–shaft correlation were calculated using MATLAB function corrcoef. Axon–axon correlations (in Extended Data Fig. 12) and bouton–shaft correlation (in Extended Data Fig. 7b) were calculated using data from each continuous imaged segment (4,000 frames by *n* boutons, frame rate: 15 Hz), then averaged over sessions on each day. For the bouton–shaft correlations of small and large peaks (in Extended Data Fig. 7d), we first identified peaks, then used data from 20 frames (5 frames before the peak position and 15 frames after peak position) to calculate the peak correlation, then averaged over all peaks in one session, then averaged over all sessions on each day. Small and large peaks were defined as peaks with an s.d. of 1–2 and 8.5–9.5, respectively.

### Nearest neighbour analysis

For each bouton, we calculated its Euclidean distances to all other boutons within the same axon, then the bouton with smallest distance were termed its nearest neighbour, and the distance was termed NND. To calculate NND distribution of the shuffled group, we randomly shuffled the bouton positions 1,000 times using MATLAB function randperm.

### Statistics

Significance testing was performed using the Wilcoxon rank sum test, Pearson correlation coefficient, one-way ANOVA, two-way ANOVA, paired *t*-test, and Kolmogorov–Smirnov test using Matlab and Microsoft Excel. Two-sided statistical tests were conducted, and data are presented as mean ± s.e.m., with all statistical tests, statistical significance values, and sample sizes described in the figure legends. *P < 0.05, **P < 0.01, ***P < 0.001; NS, not significant. All source data are included in the source data table. Sample size was first estimated on the basis of our lab's previous established protocols and previous publications. After we had an estimate of the data variance and distribution, power analysis was used to confirm that our estimated sample sizes were sufficient. We performed power analyses using the formula *N* = (*ZS*/*E*)^2^, where *Z* is the statistical significance level, *S* is the standard deviation and *E* is the margin of error. Mice were randomly assigned to control and training groups. All experiments were repeated in a minimum of three cohorts. All attempts at replication were successful. Experimenters were not blinded to experimental conditions during data collection since all mice had to progress through early and late phases of learning, which are the main condition used for comparison. Experimenters were blinded to experimental conditions during data analysis.

### Reporting summary

Further information on research design is available in the Nature Portfolio Reporting Summary linked to this article.

## Online content

Any methods, additional references, Nature Portfolio reporting summaries, source data, extended data, supplementary information, acknowledgements, peer review information; details of author contributions and competing interests; and statements of data and code availability are available at 10.1038/s41586-025-09336-w.

## Supplementary information

Reporting Summary

Supplementary Video 1 Head-fixed lever pushing behaviour. This video shows a head-fixed mouse performing the cued lever-pushing task during the late stage of learning. Blue patch in the video: time of presentation of the auditory cue. Top: cue onset time (blue bar); Middle: lever-pushing movement trajectory; Bottom: time of reward delivery (red bar).

Supplementary Video 2 GCaMP6 imaging of corticostriatal axons and boutons. This video shows the activity of corticostriatal axons and boutons in the same FOV imaged by using AAV-GCaMP6s from a mouse performing the cued lever-pushing task during the early (left) and late (right) stages of learning. Top: cue onset time (blue bar); Middle: lever-pushing movement trajectory; Bottom: time of reward delivery (red bar).

Supplementary Video 3 Heterogeneous responses at different boutons formed on the same axon. This video shows the calcium transients of different boutons and axonal shaft from the same axon. Top: averaged FOV showing the axon morphology with clear bouton structures. Middle: raw calcium imaging video showing the calcium activity of the axon and boutons. Bottom: Extracted and z-scored calcium traces of different boutons within the same axon and the axonal shaft. Numbers 1-6 marks the boutons from where the calcium transients were illustrated.

Supplementary Video 4 Longitudinal Ca^2+^ imaging of the same set of boutons formed on the same axon. This video shows the calcium transients of boutons formed on the same axon during the early (left) and late (right) stages of learning. Top: averaged FOV showing the morphology of the same axon and boutons. Middle: raw calcium imaging video showing the calcium activity of the axon and boutons. Bottom: Extracted and z-scored calcium traces of different boutons (labelled 1-6) within the same axon. Red lines indicate the onset of rewarded lever movements (RM); Blue lines indicate unrewarded lever movements (UM). Hollow circles highlight heterogeneous RM-related responses within different boutons on the same axon. Solid circles highlight homogeneous RM-related responses.

Supplementary Video 5 GCaMP6 imaging of thalamostriatal axons and boutons. This video shows the activity of thalamostriatal axons and boutons in the same FOV imaged by using AAV-GCaMP6s from a mouse performing the cued lever-pushing task during the late stage of learning. Top: cue onset time (blue bar); Middle: lever-pushing movement trajectory; Bottom: time of reward delivery (red bar).

## Source data

Source Data Fig. 1

Source Data Fig. 2

Source Data Fig. 3

Source Data Fig. 4

Source Data Extended Data Fig. 1

Source Data Extended Data Fig. 2

Source Data Extended Data Fig. 3

Source Data Extended Data Fig. 4

Source Data Extended Data Fig. 5

Source Data Extended Data Fig. 6

Source Data Extended Data Fig. 7

Source Data Extended Data Fig. 8

Source Data Extended Data Fig. 9

Source Data Extended Data Fig. 10

Source Data Extended Data Fig. 11

Source Data Extended Data Fig. 12

Source Data Extended Data Fig. 13

Source Data Extended Data Fig. 14

Source Data Extended Data Fig. 15

## Figures and Tables

**Extended Data Table 1:**
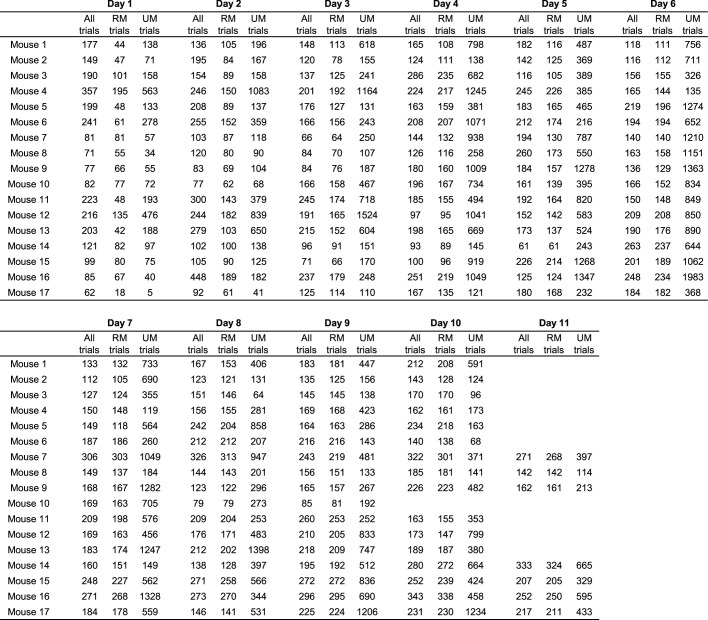
Number of all trials, RM, and UM for each mouse

List of number of all cued trials (all trials), rewarded movements during the trial period (RM trials), and unrewarded movements, including pushes failing to exceed the threshold or during the ITI, (UM trials) for all mice and training days.

## Data Availability

All source data are provided with this paper. Imaging and behaviour datasets have been deposited at Zenodo (10.5281/zenodo.15632295 (ref. ^58^)). In addition, all datasets, protocols and key lab materials used and generated in this study are listed in a key resource table alongside their public persistent identifiers at Zenodo (10.5281/zenodo.15179158 (ref. ^59^)). Source data are provided with this paper.

## Code availability

The code used and generated in this study has been deposited at Zenodo (10.5281/zenodo.15183635 (ref. ^60^)).
