# Supplementary material for: Remodelling of corticostriatal axonal boutons during motor learning
Source: Nature. 2025 Jul 30;646(8083):143–51. doi: 10.1038/s41586-025-09336-w (PMC12488484; doi:10.1038/s41586-025-09336-w)
Supplement: Supplementary file 1 — Reporting Summary [file 41586_2025_9336_MOESM1_ESM.pdf]

nature portfolio

Corresponding author(s): Jun Ding

Last updated by author(s): Jun 13, 2025

Reporting Summary

Nature Portfolio wishes to improve the reproducibility of the work that we publish. This form provides structure for consistency and transparency in reporting. For further information on Nature Portfolio policies, see our Editorial Policies and the Editorial Policy Checklist.

Statistics

For all statistical analyses, confirm that the following items are present in the figure legend, table legend, main text, or Methods section.

n/a	Confirmed	
☐	☒	The exact sample size (*n*) for each experimental group/condition, given as a discrete number and unit of measurement
☐	☒	A statement on whether measurements were taken from distinct samples or whether the same sample was measured repeatedly
☐	☒	The statistical test(s) used AND whether they are one- or two-sided<br>*Only common tests should be described solely by name; describe more complex techniques in the Methods section.*
☐	☒	A description of all covariates tested
☐	☒	A description of any assumptions or corrections, such as tests of normality and adjustment for multiple comparisons
☐	☒	A full description of the statistical parameters including central tendency (e.g. means) or other basic estimates (e.g. regression coefficient) AND variation (e.g. standard deviation) or associated estimates of uncertainty (e.g. confidence intervals)
☐	☒	For null hypothesis testing, the test statistic (e.g. *F*, *t*, *r*) with confidence intervals, effect sizes, degrees of freedom and *P* value noted<br>*Give P values as exact values whenever suitable.*
☒	☐	For Bayesian analysis, information on the choice of priors and Markov chain Monte Carlo settings
☒	☐	For hierarchical and complex designs, identification of the appropriate level for tests and full reporting of outcomes
☐	☒	Estimates of effect sizes (e.g. Cohen's *d*, Pearson's *r*), indicating how they were calculated

Our web collection on statistics for biologists contains articles on many of the points above.

Software and code

Policy information about availability of computer code

Data collection	Two-photon imaging data were acquired using ThorImageLS 4.3 (Thorlabs). Mouse behavior data were collected using (Labview 2019).
Data analysis	Data were analyzed using ImageJ (1.42o) software, Adobe Photoshop (CC), subpixel image registration algorithm (V1.1.0.0, Guizar-Sicairos et al., 2008), custom MATLAB (2020b) code, and Microsoft Excel (Microsoft 365). The code used and generated in this study has been deposited at Zenodo (doi.org/10.5281/zenodo.15183635).

For manuscripts utilizing custom algorithms or software that are central to the research but not yet described in published literature, software must be made available to editors and reviewers. We strongly encourage code deposition in a community repository (e.g. GitHub). See the Nature Portfolio guidelines for submitting code & software for further information.

Data

Policy information about availability of data

All manuscripts must include a data availability statement. This statement should provide the following information, where applicable:

- Accession codes, unique identifiers, or web links for publicly available datasets
- A description of any restrictions on data availability
- For clinical datasets or third party data, please ensure that the statement adheres to our policy

All source data are provided with this paper. Imaging and behavior datasets have been deposited at Zenodo (doi.org/10.5281/zenodo.15632295). In addition, all

datasets, protocols, and key lab materials used and generated in this study are listed in a Key Resource Table alongside their public persistent identifiers at Zenodo (doi.org/10.5281/zenodo.15179158).

Human research participants

Policy information about studies involving human research participants and Sex and Gender in Research.

Reporting on sex and gender	N/A
Population characteristics	N/A
Recruitment	N/A
Ethics oversight	N/A

Note that full information on the approval of the study protocol must also be provided in the manuscript.

Field-specific reporting

Please select the one below that is the best fit for your research. If you are not sure, read the appropriate sections before making your selection.

☒ Life sciences ☐ Behavioural & social sciences ☐ Ecological, evolutionary & environmental sciences

For a reference copy of the document with all sections, see nature.com/documents/nr-reporting-summary-flat.pdf

Life sciences study design

All studies must disclose on these points even when the disclosure is negative.

Sample size	Sample size was first estimated based on our lab's previous established protocols, and previous publications. After we have an estimate on the data variance, and distribution, power analysis was used to confirm that our estimated sample sizes were sufficient. We performed power analyses, using the formula N = [Z*S / E]^2, where Z is the statistical significance level, S is the standard deviation, and E is the margin of error.
Data exclusions	For imaging experiments, animals were excluded if (1) Raw calcium movies showed large motion artifacts. (2) GCaMP6 or EGFP expression were too dense or too sparse expression for imaging analysis. For analyzing activities of different boutons formed on the same axon, only those had clear axon and bouton morphology were included.
Replication	All experiments were repeated in a minimum of three cohorts. All attempts at replication were successful.
Randomization	Mice were randomly assigned to control or training group.
Blinding	Experimenters were not blinded to experimental conditions during data collection since all mice had to progress through early and late phases of learning, which are the main conditions used for comparison. Experimenters were blinded to experimental conditions during data analysis.

Reporting for specific materials, systems and methods

We require information from authors about some types of materials, experimental systems and methods used in many studies. Here, indicate whether each material, system or method listed is relevant to your study. If you are not sure if a list item applies to your research, read the appropriate section before selecting a response.

Materials & experimental systems

n/a	Involved in the study	
☒	☐	Antibodies
☒	☐	Eukaryotic cell lines
☒	☐	Palaeontology and archaeology
☐	☒	Animals and other organisms
☒	☐	Clinical data
☒	☐	Dual use research of concern

Methods

n/a	Involved in the study	
☒	☐	ChIP-seq
☒	☐	Flow cytometry
☒	☐	MRI-based neuroimaging

Animals and other research organisms

Policy information about studies involving animals; ARRIVE guidelines recommended for reporting animal research, and Sex and Gender in Research

Laboratory animals	All animals were kept at a 12hr:12hr light/dark cycle at a room temperature of 22ºC with humidity control (30-70%). WT mice (C57BL/6J, aged 7 weeks - 6 months) of both males and females from The Jackson Laboratory were used in the present study.
Wild animals	The study did not involve wild animals.
Reporting on sex	Both male and female mice were used in this study at an approximately 50/50 ratio.
Field-collected samples	The study did not involve samples collected from the field.
Ethics oversight	All experiments were performed in accordance with protocols approved by the Stanford University Animal Care and Use Committee, in accordance with the National Institutes of Health's Guide for the Care and Use of Laboratory Animals.

Note that full information on the approval of the study protocol must also be provided in the manuscript.
